# A national recruitment strategy for HIV-serodiscordant partners living in Canada for the Positive Plus One study: a mixed-methods study

**DOI:** 10.1186/s12889-022-13153-5

**Published:** 2022-04-26

**Authors:** Min Xi, Sandra Bullock, Joshua B. Mendelsohn, James Iveniuk, Veronika Moravan, Ann N. Burchell, Darrell H. S. Tan, Amrita Daftary, Tamara Thompson, Bertrand Lebouché, Laura Bisaillon, Ted Myers, Liviana Calzavara

**Affiliations:** 1 KITE Research Institute, Toronto Rehabilitation Institute, University Health Network, Toronto, ON M5G 2A2 Canada; 2 Institute of Health Policy, Management and Evaluation, University of Toronto, Toronto, ON M5T 3M6 Canada; 3 Dalla Lana School of Public Health, University of Toronto, 155 College St, Toronto, ON M5T 3M7 Canada; 4 College of Health Professions, Pace University, New York, 10038 USA; 5 Academic Research Centers, NORC at the University of Chicago, Chicago, IL 60637 USA; 6 VM Stats, Toronto, ON M5A 4R3 Canada; 7 MAP Centre for Urban Health Solutions, Li Ka Shing Knowledge Institute, St Michael’s Hospital, Unity Health Toronto, Toronto, ON M5B 1W8 Canada; 8 Department of Family and Community Medicine, Faculty of Medicine, University of Toronto, Toronto, ON M5G 1V7 Canada; 9 Division of Infectious Diseases, St Michael’s Hospital, Unity Health Toronto, Toronto, ON M5B 1W8 Canada; 10 School of Global Health and Dahdaleh Institute for Global Health Research, York University, Toronto, ON M3J 1P3 Canada; 11 Faculty of Health Sciences, Douglas College, Coquitlam, BC V3B 7X3 Canada; 12 Department of Family Medicine, Faculty of Medicine, McGill University, Montreal, QC H3S 1Z1 Canada; 13 Department of Medicine, Division of Infectious Diseases and Chronic Viral Illness Service, McGill University Health Centre Montreal, Montreal, QC H4A 3J1 Canada; 14 Center for Outcomes Research and Evaluation, Research Institute of the McGill University Health Centre, Montreal, QC H4A 35S Canada; 15 Department of Health and Society, University of Toronto Scarborough, Toronto, ON M1C 1A4 Canada

**Keywords:** HIV, Study method, Recruitment, HIV-serodiscordant couple, Canada, Dyad, Study design

## Abstract

**Background:**

With the recent shift in focus to addressing HIV risk within relationships and couple-based interventions to prevent HIV transmission, successful recruitment of individuals involved in HIV-serodiscordant relationships is crucial. This paper evaluates methods used by the Positive Plus One (PP1) study to recruit and collect data on a diverse national sample of dyads and individuals involved in current or past HIV-serodiscordant relationships, discusses the strengths and limitations of the recruitment approach, and makes recommendations to inform the interpretation of study results and the design of future studies.

**Methods:**

PP1 used a multi-pronged approach to recruit adults involved in a current or past HIV-serodiscordant relationship in Canada from 2016 to 2018 to complete a survey and an interview. Upon survey completion, index (first recruited) partners were invited to recruit their primary current HIV-serodiscordant partner. We investigated participant enrollment by recruitment source, participant-, relationship-, and dyad-level sociodemographic characteristics, missing data, and correlates of participation for individuals recruited by their partners.

**Results:**

We recruited 613 participants (355 HIV-positive; 258 HIV-negative) across 10 Canadian provinces, including 153 complete dyads and 307 individuals who participated alone, and representing 460 HIV-serodiscordant relationships. Among those in current relationships, HIV-positive participants were more likely than HIV-negative participants to learn of the study through an ASO staff member (36% v. 20%, *p* < 0.001), ASO listserv/newsletter (12% v. 5%, *p* = 0.007), or physician/staff at a clinic (20% v. 11%, *p* = 0.006). HIV-negative participants involved in current relationships were more likely than HIV-positive participants to learn of the study through their partner (46% v. 8%, *p* < 0.001). Seventy-eight percent of index participants invited their primary HIV-serodiscordant partner to participate, and 40% were successful. Successful recruitment of primary partners was associated with longer relationship duration, higher relationship satisfaction, and a virally suppressed HIV-positive partner.

**Conclusions:**

Our findings provide important new information on and support the use of a multi-pronged approach to recruit HIV-positive and HIV-negative individuals involved in HIV-serodiscordant relationships in Canada. More creative strategies are needed to help index partners recruit their partner in relationships with lower satisfaction and shorter duration and further minimize the risk of “happy couple” bias.

## Background

As of 2018, approximately 62,050 individuals in Canada were estimated to be living with HIV, with an incidence of 5.6 per 100,000 individuals per year [1]. Approximately 85% of diagnosed individuals were receiving treatment, of whom 94% reported viral suppression [2]. Since the introduction of combination antiretroviral therapy (ART) and adoption of Undetectable = Untransmittable (U=U), HIV has become a chronic and manageable disease in resource-rich settings where individuals reporting durable viral suppression had comparable life expectancy to individuals in matched controls [3–6]. A Swiss study found that the life expectancy of individuals living with HIV at the age of 20 increased from 11.8 years during the monotherapy era (i.e., 1988–1991) to 54.9 years in the early stages of the ART era (i.e., 2006–2013) [3].

While the incidence of HIV has stabilized in Canada and elsewhere following the introduction of ART, the number of individuals living with HIV in Canada is expected to grow, and with it, the number of primary HIV-serodiscordant couples [7, 8]. Data from Sub-Saharan Africa suggest that approximately 50 to 75% of HIV-positive individuals are involved in HIV-serodiscordant relationships [9, 10]. Previous studies conducted in Zambia and in the USA have shown that 60–94 and 68% of incident HIV cases among heterosexual individuals and men who have sex with men (MSM), respectively, were attributable to primary sex partners [10, 11]. In recent years, the focus has shifted to addressing HIV risk within relationships and couple-based interventions to promote safer sex and prevent HIV transmission [12–14]. However, there are gaps in knowledge regarding experiences of HIV-serodiscordant couples and their management of HIV transmission risk in the ART era, including the quality and extent of supportive services from the perspective of both HIV-positive and HIV-negative partners in a relationship and, given partners’ differential experiences and perceptions, of the couple as a unit [15–22]. In a recent scoping review, our team identified gaps in the evidence available in Canada, particularly among those involved in stable, long-term, or primary serodiscordant relationships [15]. Many studies recruited participants from HIV clinics and other clinical settings, missing HIV-negative partners who did not require direct HIV care services and potentially missing HIV-positive individuals not linked to or retained in care [15, 23–25]. Data were lacking on HIV-serodiscordant couples within key populations including transgender, Indigenous, and immigrant communities, including those from HIV-endemic areas, who may experience significant barriers to HIV education, care, and supportive resources [15].

Dyadic studies (i.e., studies involving both partners in a relationship) can provide a better understanding of the interplay of individual and dyadic experiences within the serodiscordant relationship [15]. For example, the HIV-negative partner’s estimate of their HIV-positive partner’s adherence to ART was shown to be a better predictor of viral suppression than the HIV-positive partner’s self-reported adherence [26, 27].

Several challenges exist in the recruitment of representative cohorts of HIV-serodiscordant dyads, introducing gaps and biases in our understanding of HIV-serodiscordant relationships. Given the lack of a means to systematically identify people in HIV-serodiscordant relationships, relationships that are undisclosed to health care providers are hidden [28]. Previous studies have reported challenges in recruiting both partners of dyads with the following characteristics: long relationships with older partners, relationships involving at least one bisexual man, relationships with higher satisfaction [29–31]. A USA study found that the successful recruitment of both partners in a dyad varied significantly by race and ethnicity, geographical region, education, and relationship type [32]. Previous studies have underscored the need to develop new and creative methods of recruiting and enrolling dyads to obtain a large, diverse sample and increase the independent participation of both partners in the dyad to limit coercion [30, 32].

To address the previously identified challenges to dyad recruitment and to design a study that was relevant, feasible, and addressed needs articulated by serodiscordant couples in the Canadian setting, the Positive Plus One (PP1) study team conducted a feasibility study among staff at AIDS Services Organizations (ASOs) and individuals in serodiscordant relationships [33]. Findings indicated the need to use multiple approaches for recruitment and survey delivery (in both English and French) to involve a national sample representing regional differences with a diverse range of sociodemographic backgrounds and sexual identities [33].

In this paper, we evaluate methods used to recruit and collect data on dyads and individuals involved in current or past HIV-serodiscordant relationships in Canada between 2016 and 2018, discuss the strengths and limitations of our approach, and make recommendations to inform the interpretation of study results and the design of future studies.

## Methods

The PP1 investigative team comprised 31 academics, clinicians, HIV/AIDS service providers, and people living with HIV from across Canada. The project aimed to understand sociodemographic characteristics, relationship satisfaction, HIV transmission risk, perceived needs and access to supportive services, and subjective experiences of individuals living within an HIV-serodiscordant relationship in Canada via an online/telephone survey followed by an in-depth telephone interview for more complex, open-ended questions. The survey was designed to take approximately 30 minutes to decrease the risk of respondent fatigue [34]. To reduce the risk of participation bias identified in previous dyad studies, PP1 used a multi-pronged recruitment strategy to survey one or both partners in a current or recently concluded serodiscordant relationship in Canada from 2016 to 2018. While our team planned for recruitment to take one year, it ultimately took two years to recruit our sample. In this paper, we describe participant enrollment from various recruitment sources. We also compare the sociodemographic characteristics of HIV-positive participants in PP1 to HIV-positive individuals included in Canada’s public health surveillance data (detailed later in Methods) and participants’ relationship characteristics by whether their partner was also recruited to the study [35]. Furthermore, we examine the proportion of missing data across the survey questions and correlates of participation for individuals recruited by partners in their relationships.

### Eligibility criteria

PP1 sought to recruit adults involved in a current or past HIV-serodiscordant relationship in Canada. Our study included individuals: (1) ≥18 years; (2) in a current or past (i.e., within two years prior to study enrollment) HIV-serodiscordant relationship; (3) living in Canada at the time of the survey and during at least part of the relationship; and (4) able to speak, read, and/or write English or French. The study definition of an HIV-serodiscordant relationship was a primary relationship where one partner was HIV-positive and the other was HIV-negative. To be considered a primary relationship, the index partner (first partner enrolled in the study) had to consider their relationship as “dating,” “together,” or “a couple.” This definition was used to discourage individuals in casual and sex only relationships from participating in the study. For polyamorous relationships, the index partner could invite one HIV-serodiscordant partner to be matched with, and other partner(s) could join, but it was not possible to match them for analysis.

### Recruitment venues and processes

**Fig. 1:**
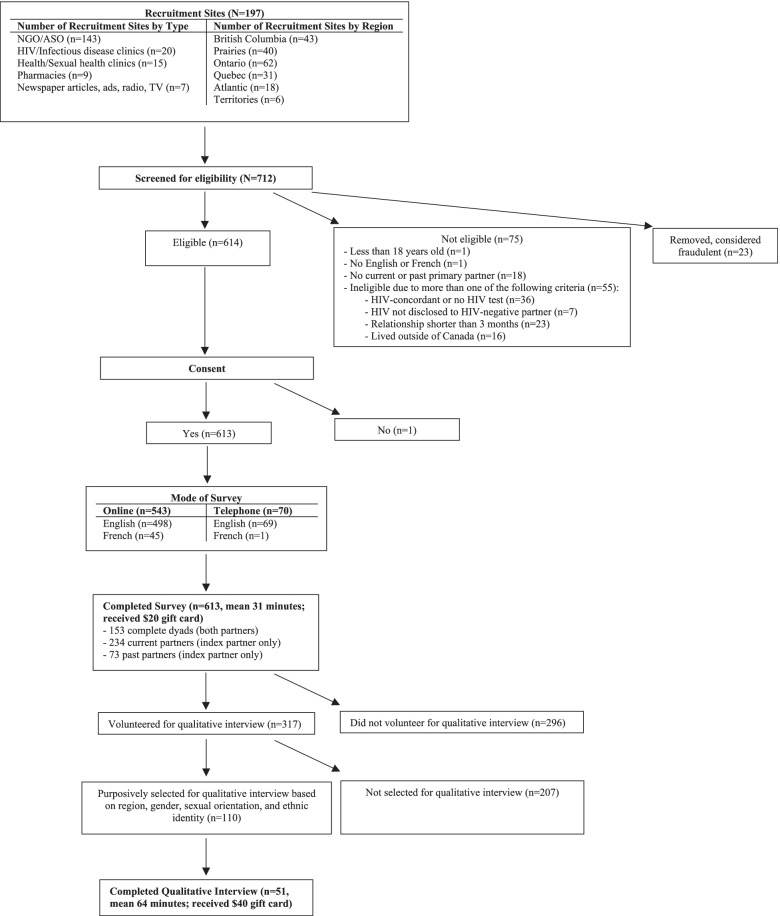
Flow chart of pathways through the study, from recruitment to completion of participation. Abbreviations: Non-governmental organization (NGO); AIDS Services Organizations (ASOs)

PP1 used a multi-pronged outreach and recruitment strategy to maximize the number of HIV-positive and HIV-negative partners in an HIV-serodiscordant relationship and HIV-serodiscordant dyads reached, thereby reducing risk of sampling bias. Table 1 and Fig. 1 show the recruitment methods as well as the different paths that participants took through the study.

**Table 1:** Recruitment sources for HIV-positive and HIV-negative PP1 participants stratified by relationship type (*N* = 613)

Recruitment Source	All Participants	Participants in Current HIV-Serodiscordant Relationships				Participants in Past HIV-Serodiscordant Relationships			
	Total (***N*** = 613)	Total (***n*** = 540)	HIV-Positive (***n*** = 312)	HIV-Negative (***n*** = 228)	***p***-value	Total (***n*** = 73)	HIV-Positive (***n*** = 43)	HIV-Negative (***n*** = 30)	***p***-value
ASO staff member	185 (30.2%)	159 (29.4%)	113 (36.2%)	46 (20.2%)	**< 0.001**	26 (35.6%)	14 (32.6%)	12 (40.0%)	0.561
ASO listserv/newsletter	56 (9.1%)	47 (8.7%)	36 (11.5%)	11 (4.8%)	**0.007**	9 (12.3%)	5 (11.6%)	4 (13.3%)	0.857
Physician/staff at clinic	96 (15.7%)	87 (16.1%)	62 (19.9%)	25 (11.0%)	**0.006**	9 (12.3%)	4 (9.3%)	5 (16.7%)	0.366
Poster, pamphlet, card	140 (22.8%)	122 (22.6%)	76 (24.4%)	46 (20.2%)	0.277	18 (24.7%)	8 (18.6%)	10 (33.3%)	0.168
Blog/website	43 (7.0%)	35 (6.5%)	19 (6.1%)	16 (7.0%)	0.643	8 (11.0%)	2 (4.7%)	6 (20.0%)	0.060
Online ad	45 (7.3%)	38 (7.0%)	26 (8.3%)	12 (5.3%)	0.178	7 (9.6%)	5 (11.6%)	2 (6.7%)	0.692
Heard from friend	57 (9.3%)	42 (7.8%)	27 (8.7%)	15 (6.6%)	0.392	15 (20.6%)	8 (18.6%)	7 (23.3%)	0.659
From partner	132 (21.5%)	129 (23.9%)	25 (8.0%)	104 (45.6%)	**< 0.001**	3 (4.1%)	1 (2.3%)	2 (6.7%)	0.567
Through news	16 (2.6%)	15 (2.8%)	7 (2.2%)	8 (3.5%)	0.366	1 (1.4%)	0	1 (3.3%)	0.431
Other^a^	30 (4.9%)	21 (3.9%)	13 (4.2%)	8 (3.5%)	0.696	9 (12.3%)	3 (7.0%)	6 (20.0%)	0.096
Don’t know	1 (0.2%)	1 (0.2%)	0	1 (0.4%)	0.420	0	0	0	–
No response	5 (0.8%)	4 (0.7%)	1 (0.3%)	3 (1.3%)	0.315	1 (1.4%)	1 (2.3%)	0	0.411

Abbreviations: *PP1* Positive Plus One Study, *ASO* AIDS Services Organization

Note: Since participants can check more than one response, percentages may add to more than 100%. Additionally, participants who checked the poster, pamphlet, or card option may have gotten these materials from different locations. It was not possible to distinguish between the different locations

^a^ This category includes community health centers, methadone clinics, investigators/conferences, pharmacies, and emails

Efforts were made to recruit participants across Canada’s 10 provinces and three territories between January 1st, 2016 and June 30th, 2018 from a broad and diverse range of sources, including: 143 non-governmental organizations (NGOs) and ASOs; 35 medical clinics (HIV, sexual health, general health clinics); and nine community pharmacies. NGOs included needle exchange programs, methadone clinics, and community centers in areas of high HIV prevalence that were not considered health clinics. We also used snowball sampling, social media (i.e., Facebook, Twitter), and television, radio, and newspaper media advertising. Most of the ASOs and clinics periodically included our study description and contact details in their online and paper newsletters. Almost all (97%) organizations approached by the team agreed to participate in recruitment efforts on behalf of the study. Staff members at various recruitment sites directly informed potential participants about the study either in-person or by telephone or email. Several sites were unable to assist with active recruitment (i.e., individually speaking with clients/patients and handing out recruitment materials) due to a variety of reasons including lack of: awareness of individual’s relationship status, staffing resources, and/or monetary compensation for doing so. At a minimum, all sites agreed to display pamphlets and posters, or send out recruitment emails for the study (passive recruitment). Internet and social media-based methods of recruitment were used to attempt to reach those not utilizing services and improve the enrollment of geographically dispersed and ethnically/racially diverse populations [36, 37]. Recruitment materials, available in English and French, directed participants to an online or telephone survey and to a bilingual study website that included study information and a link to the online survey. Snowball sampling provided an online vehicle encouraging participating study members to share the study details and website with up to three friends or acquaintances in hopes of recruiting individuals unlinked to ASOs or HIV care.

### Index partners

Eligible adults in current or past HIV-serodiscordant relationships were invited to participate in the online survey after eligibility screening and informed consent. The survey lasted a mean of 31 minutes (SD: 15). The survey was administered separately to individual partners by choice of telephone (toll-free) or online,^1^ in English or French. The online survey was accessible from any location with internet connection; IP addresses were not collected. Participants electing live telephone interview were advised to complete the survey in a private location or to schedule an interview appointment for a time when they could. To mitigate social desirability bias, participants were specifically asked not to complete the survey with the assistance of another person, especially their partner.

### Partner recruitment

We used a snowball sampling approach to recruit dyad partners. Index partners were invited to recruit their primary HIV-serodiscordant partner directly following survey completion and were provided a reminder 1 week later if they were willing to share their own email address. To facilitate the linkage of partners in a relationship, the index partner provided a unique alpha-numeric code to share with their partner, that could link partner surveys. In a limited number of cases, dyadic partners were matched manually if they completed the survey at the same time or if they forgot their code and contacted the study office. Linkage facilitated the generation of dyadic outcomes including combined and difference scores for dyadic analyses. The index partner was eligible to participate regardless of whether their partner chose to enrol. Individuals who were part of a past HIV-serodiscordant relationship were not asked to invite their previous partner(s) to the study.

All participants who completed the survey were invited to volunteer for participation in a 60–90 minute qualitative semi-structured interview to provide in-depth understanding of lived experiences. A diverse sub-sample of volunteers was purposively selected and invited to complete the interview. Practices used to manage personal information were outlined on the study website, and in the consent form. To ensure anonymity of survey responses, participants who chose to receive a gift card were forwarded upon completion of the survey to a separate unlinked form to provide their mailing address if they chose to receive a token-of-appreciation (i.e., $20 gift card) for their time and participation. Personal information (name, phone number, postal and email addresses) used to make contact for the qualitative interview and to send gift cards were stored in a separate database that could only be linked with survey responses by the Research Coordinator and Principal Investigator; this link was destroyed upon completion of data collection.

### Data quality considerations

Collecting data using telephone and online surveys is an effective way to reach a diverse national sample, particularly to reach individuals outside of major urban centres [36, 37]. However, online research comes with its own limitations. Online surveys, especially those offering incentives to participate, are challenged by duplicate and fraudulent entries [38–41]. Although most participants provide high quality information, an attempt was made to prevent, detect, and exclude invalid or falsified surveys, as they could introduce non-trivial amounts of measurement error or social bias to the study. We took the following steps to reduce this risk: (1) gift cards were provided via Canada Post, requiring a name and full Canadian mailing address [41]; and (2) ineligibility feedback was not provided to avoid making it too easy for individuals to adjust responses and fraudulently re-take the survey. Surveys were flagged for manual follow-up if they met any of the following criteria: (1) short completion time (< 8 minutes); (2) cluster of several non-eligible attempts were made to respond to the survey from the same device-type, time zone, and city; (3) several surveys completed at a physical address within a small, localized region; (4) random/illogical response patterns; (5) high rate of missing and/or don’t know data; and/or (6) complete duplication (including gift card name/address) of an already completed survey. Once flagged, we conducted a case-by-case manual review and 23 were excluded, each meeting several of the listed criteria.

### Survey measures

Data were collected on the participant and partner’s sociodemographic information; relationship dynamics; relationship satisfaction; sexual satisfaction within and outside of the relationship; HIV management including use, attitudes, and beliefs surrounding condoms, pre- and post-exposure prophylaxis (PEP and PrEP); health status, use of ART, and viral suppression; injection drug use; HIV disclosure to friends, family, and medical personnel; social support and HIV-support needs.

### Surveillance data

We used PHAC HIV surveillance data collected between 1985 and 2016 [35]. These data included all reported diagnosed cases since the beginning of the HIV epidemic. Notably, the early HIV epidemic was centered predominantly among individuals who identified as White MSM, a large proportion of whom may no longer be alive [42]. The current HIV epidemic in Canada has involved more individuals of colour and more individuals who identified as heterosexual [42]. Although it may be more appropriate to compare our participant demographics to current HIV prevalent cases in Canada, these data were not publicly available.

PHAC data were derived from the national HIV/AIDS Surveillance System (HASS), the data collected through immigration medical screening for HIV by Immigration, Refugees and Citizenship Canada (IRCC), and the Canadian Perinatal HIV Surveillance Program (CPHSP). The HASS monitors HIV cases in Canada by collating non-nominal data voluntarily submitted by all Canadian provinces and territories. It should be noted that race/ethnicity data were not available for any province or territory prior to 1998 and remained unavailable for Quebec and British Columbia in the 2016 PHAC surveillance report [35]. Additionally, race/ethnicity were only reported by Ontario after 2009 [35]. Since race/ethnicity data were only reported for approximately 50% of HIV cases, these data may not be fully representative of people living with HIV in Canada [35].

### Statistical analysis

Analyses were carried out in SAS (Studio 9.4; SAS Institute Inc.), OpenEpi [43], and R (v. 4.0.4; R Core Team 2021). We used descriptive statistics to examine the sources from which individual participants and dyads learned of the study; demographic characteristics of individual study participants; dyad- and relationship-level sociodemographic and HIV-related characteristics; and relationship and sexual satisfaction within the dyad. Counts and proportions were calculated for categorical variables whereas means/medians, standard deviations, and ranges were calculated for continuous variables. The sample distribution of people living with HIV in a current or past HIV-serodiscordant relationship was compared with 1985 to 2016 PHAC surveillance data using chi-square tests and 95% confidence intervals on gender, sexual orientation, ethno-racial identity, region, and age at HIV diagnosis. Since 95% confidence intervals were not provided for PHAC data, confidence intervals were calculated using the Newcombe-Wilson method, assuming the PHAC data were normally distributed [44]. Missing data were not included in this comparison analysis. Sociodemographic and HIV-related characteristics of dyads and relationships represented by one partner were compared. Chi-square tests were used for categorical variables, t-tests for continuous variables, and Wilcoxon rank sum tests for ordinal variables. Associations between HIV status, sociodemographic variables, relationship satisfaction, sexual satisfaction, and the proportion of individuals in a current HIV-serodiscordant relationship who recruited their HIV-serodiscordant partner to the study were examined. Chi-square tests and 95% confidence intervals were used for categorical variables and t-tests were used for continuous variables. Two-sided Fisher’s exact tests were used for categorical and dichotomous variables with an expected cell count of less than five. All testing was two-sided, and we used an alpha level of .05 for all statistical tests.

## Results

We recruited 613 participants (355 HIV-positive; 258 HIV-negative) over two years, including 540 participants in a current HIV-serodiscordant relationship at the time of the study and 73 participants from past relationships that ended within two years prior to survey completion. At the dyad level, 306 participants were recruited from 153 relationships that included both partners (i.e., complete dyads); 307 individuals participated without their current partner. In total 460 relationships were represented.

### Recruitment sources

Table 1 describes the method(s) through which the participant became aware of the study by their current or past relationship status. A plurality of participants involved in a current relationship learned of the study from ASO staff members (29%), while almost a quarter learned of the study from their partner (24%) or a poster, pamphlet, or card (23%). Participants involved in past relationships mainly heard of the study through an ASO staff member (36%), a poster, pamphlet, or card (25%), or a friend (21%). Among those in current relationships, HIV-positive participants were more likely than HIV-negative participants to learn of the study through an ASO staff member (36% v. 20%, *p* < 0.001), an ASO listserv/newsletter (12% v. 5%, *p* = 0.007), or a physician or staff at a clinic (20% v. 11%, *p* = 0.006). HIV-negative participants involved in current relationships were more likely than HIV-positive participants to learn of the study through their partner (46% v. 8%, *p* < 0.001). These associations were not detected in past relationships, potentially due to the small number of participants involved in a past HIV-serodiscordant relationship in our study.

### Participant demographics

The majority of participants completed the survey online (89%) and in English (93%; Table 2). The mean ages of participants in current and past relationships were similar (43 (SD:12) v. 41 (SD:12), *p* = 0.204). Similar proportions of HIV-positive individuals were recruited from current and past relationships (58% v. 59%, *p* = 0.855). Most participants in both types of relationships resided in Ontario (58% v. 58%, *p* = 0.162) and identified as white (67% v. 66%, *p* = 0.771). Participants involved in a current HIV-serodiscordant relationship reported higher education levels (i.e., beyond secondary school; 69% v. 57%, *p* = 0.007) and longer relationship duration (i.e., 10 years or more; 30% v. 6%, *p* < 0.001) compared to those involved in past relationships. A larger proportion of participants in current relationships identified as gay men (48% v. 29%, *p* = 0.003) and had not been involved in a previous HIV-serodiscordant relationship prior to this study (66% v. 47%, *p* = 0.009) compared to participants in past relationships. Approximately a third of participants reported an annual income of $20,000 to $49,999 and half of participants had a full-time job.

**Table 2:** Sociodemographic characteristics of all PP1 participants by current and past HIV-serodiscordant relationship

Characteristic	Total N (%)	Current Relationship n (%)	Past Relationship n (%)	***p***-value
**All**	**613**	**540**	**73**	
**Age, Mean (SD)**	42.6 (11.9)	42.8 (11.9)	40.9 (12.0)	0.204
**Survey format**	**613**	**540**	**73**	0.795
Web	543 (88.6)	479 (88.7)	64 (87.7)	
Phone	70 (11.4)	61 (11.3)	9 (12.3)	
**Language**	**613**	**540**	**73**	0.805
English	567 (92.5)	500 (92.6)	67 (91.8)	
French	46 (7.5)	40 (7.4)	6 (8.2)	
**HIV status**	**613**	**540**	**73**	0.855
Positive	355 (57.9)	312 (57.8)	43 (58.9)	
Negative	258 (42.1)	228 (42.2)	30 (41.1)	
**Province**	**609**	**536**	**73**	0.162
British Columbia	70 (11.5)	65 (12.1)	5 (6.9)	
Prairies	86 (14.1)	72 (13.3)	14 (19.2)	
Ontario	351 (57.6)	309 (57.7)	42 (57.5)	
Quebec	66 (10.8)	55 (10.3)	11 (15.1)	
Atlantic	36 (5.9)	35 (6.5)	1 (1.4)	
Territories	0	0	0	
**Gender**	**612**	**539**	**73**	**0.006**
Man	432 (70.6)	392 (72.7)	40 (54.8)	
Woman	166 (27.1)	136 (25.2)	30 (41.1)	
Other^a^	14 (2.3)	11 (2.0)	3 (4.1)	
**Gender by sexuality**	**613**	**540**	**73**	**0.003**
Gay man	281 (45.8)	260 (48.1)	21 (28.8)	
Heterosexual woman	135 (22.0)	109 (20.2)	26 (35.6)	
Heterosexual man	109 (17.8)	97 (18.0)	12 (16.4)	
Other^b^	88 (14.4)	74 (13.7)	14 (19.2)	
**Race/ethnicity**	**604**	**531**	**73**	0.771
White	401 (66.4)	353 (66.5)	48 (65.8)	
Black	47 (7.8)	43 (8.1)	4 (5.5)	
Hispanic	49 (8.1)	40 (7.5)	9 (12.3)	
Indigenous	49 (8.1)	44 (8.3)	5 (6.8)	
Other^c^	58 (9.6)	51 (9.6)	7 (9.6)	
**Education**	**609**	**537**	**72**	**0.007**
Less than secondary school diploma	72 (11.8)	59 (11.0)	13 (18.1)	
Secondary school diploma	127 (20.9)	109 (20.3)	18 (25.0)	
Beyond secondary school	410 (67.3)	369 (68.7)	41 (56.9)	
**Income**	Unavailable	**535**	Unavailable	–
No income		16 (3.0)		
Less than $10,000		50 (9.4)		
$10,000 - $19,999		127 (23.7)		
$20,000 - $49,999		173 (32.3)		
$50,000 - $79,999		82 (15.3)		
$80,000 or more		79 (14.8)		
Don’t know		8 (1.5)		
**Employment**^**d**^	Unavailable	**538**	Unavailable	–
Full-time		272 (50.6)		
Part-time		66 (12.3)		
On disability		116 (21.6)		
Volunteer		44 (8.2)		
Retired		36 (6.7)		
Student		33 (6.1)		
Family work		11 (2.0)		
Informal/street-related work		8 (1.5)		
Unemployed, seeking work		42 (7.8)		
Unemployed, not seeking work		11 (2.0)		
Other^e^		6 (1.2)		
**Relationship duration**	**613**	**540**	**73**	**< 0.001**
< 1 year	82 (13.4)	63 (11.7)	19 (26.0)	
1–2 years	143 (23.3)	116 (21.5)	27 (37.0)	
3–5 years	142 (23.2)	124 (23.0)	18 (24.7)	
6–9 years	79 (12.9)	74 (13.7)	5 (6.9)	
10 years or more	167 (27.4)	163 (30.2)	4 (5.5)	
**In a serodiscordant relationship before this study**	**607**	**535**	**72**	**0.009**
No	385 (63.4)	351 (65.6)	34 (47.2)	
Yes	195 (32.1)	161 (30.1)	34 (47.2)	
Don’t know	27 (4.4)	23 (4.3)	4 (5.6)	

Abbreviations: *PP1* Positive Plus One Study

^a^ This category includes participants who identified as a “trans man,” “trans woman,” “two-spirited man,” and participants who did not identify as any gender

^b^ This category includes participants who identified as a “trans man,” “trans woman,” “two-spirited man,” “two-spirited woman,” “lesbian,” “bisexual man,” “bisexual woman,” and participants who did not identify as any gender and/or sexual orientation, did not believe in labels for gender and sexual orientation, or did not respond

^c^ This category includes participants who identified as Arab/West Asian, East Asian, South Asian, South-East Asian, and other (the most common other response was “mixed”)

^d^ Participants were able to select all response options that applied to them (i.e., participants were able to select more than one response option)

^e^ Participants were not asked to specify what they meant by “other”

### HIV-positive participants

Among HIV-positive participants in PP1, 312 were in a current relationship and 43 had been in a past relationship. Of the 312 participants in current relationships, 250 (80%) were index partners and 62 (20%) were invited to the study by their index partner. To examine the national representativeness of our HIV-positive participants, we compared the sociodemographic characteristics of all 355 HIV-positive participants enrolled in PP1 to HIV-positive individuals included in the 1985–2016 PHAC HIV surveillance database. Compared with 1985–2016 PHAC surveillance data, PP1 under-represented HIV-positive individuals who were aged 30 years or older at the time of their HIV diagnosis (51% v. 73%, *p* < 0.001), individuals who identified as men (67% v. 80%, *p* < 0.001), Black (7% v. 20%, *p* < 0.001), Indigenous (10% v. 25%, *p* < 0.001), and individuals who resided in British Columbia (11% v. 18%, *p* < 0.001), Alberta (5% v. 8%, *p* < 0.001), and Quebec (12% v. 23%, *p* < 0.001) and the three territories (Table 3).

**Table 3:** Characteristics of PP1 HIV-positive participants compared to HIV-positive individuals in the 1985–2016 PHAC surveillance data^a^

Demographic Characteristic	Positive Plus One Data (***N*** = 355)			PHAC Data (***N*** = 84,409)			***p***-value
	N	%	95% CI	N	%	95% CI	
**Age at diagnosis**	**348**	100	–	**79,874**	100	–	**< 0.001**
< 15	9	2.6	0.9–4.3	657	0.8	0.8–0.9	
15–19	14	4.0	2.0–6.1	1323	1.7	1.6–1.8	
20–29	148	42.5	37.3–47.8	19,952	25.0	24.7–25.3	
30–39	113	32.5	27.5–37.4	30,083	37.7	37.3–38.0	
40–49	53	15.2	11.4–19.0	18,394	23.0	22.7–23.2	
≥ 50	11	3.2	1.3–5.0	9465	11.8	11.6–12.1	
Unknown/Missing	7^b^	–	–	4535^c^	–	–	
**Gender**	**355**	100	–	**80,134**	100		**< 0.001**
Male	239	67.3	62.4–72.2	64,127	80.0	79.8–80.3	
Female	108	30.4	25.6–35.2	14,758	18.4	18.2–18.7	
Sex not reported/	4	1.1	0.0–2.3	1249	1.6	1.5–1.7	
Transexual/Transgender							
Other^d^	4	1.1	0.0–2.3	–	–	–	
**Sexual orientation**^**e**^	**348**	100	–	**45,554**	100	–	**< 0.001**
Heterosexual	141	40.5	35.3–45.7	19,316^g^	42.4	42.0–42.9	
MSM	195^f^	56.0	50.8–61.3	26,238^h^	57.6	57.1–58.1	
Other	12	3.5	1.5–5.4	–	–	–	
Missing	7	–	–	34,580^i^	–	–	
**Race/ethnicity**^**j**^	**348**	100	–	**12,453**	100	–	**< 0.001**
White	220	63.2	58.1–68.3	5528	44.4	43.5–45.3	
Black	25	7.2	4.5–9.9	2448	19.7	19.0–20.4	
Indigenous^k^	34	9.8	6.6–12.9	3076	24.7	24.0–25.5	
Hispanic	33	9.5	6.4–12.6	422	3.4	3.1–3.7	
East/Southeast Asian	11	3.2	1.3–5.0	528	4.2	3.9–4.6	
South Asian/West Asian/Arab	10	2.9	1.1–4.6	287	2.3	2.1–2.6	
Other	15	4.3	2.2–6.5	164	1.3	1.1–1.5	
Missing	7	–	–	31,145^l^	–	–	
**Region**	**352**	100	–	**84,409**	100	–	**< 0.001**
British Columbia	37	10.5	7.3–13.7	15,529	18.4	18.1–18.7	
Alberta	19	5.4	3.0–7.8	6981	8.3	8.1–8.5	
Saskatchewan	22	6.3	3.7–8.8	2341	2.8	2.7–2.9	
Manitoba	8	2.3	0.7–3.8	2350	2.8	2.7–2.9	
Ontario	205	58.2	53.1–63.4	36,319	43.0	42.7–43.4	
Quebec	41	11.6	8.3–15.0	19,111	22.6	22.4–22.9	
Atlantic	20	5.7	3.3–8.1	1652	2.0	1.9–2.1	
Territories	0	–	–	126	0.1	0.1–0.2	
Missing	3	–	–	0	–	–	

Abbreviations: *PP1* Positive Plus One, *PHAC* Public Health Agency of Canada

^a^ PHAC surveillance data include a lot of individuals who are no longer alive, primarily those who were infected during the pre-ART era. It should be noted that the early Canadian HIV epidemic primarily involved individuals who identified as White MSM whereas more recently the Canadian HIV epidemic has involved more individuals of colour and individuals who identified as heterosexual

^b^ This category includes missing responses in Positive Plus One data

^c^ This category includes “unknown” and “not reported” in 1985–2016 PHAC data

^d^ This category includes “two-spirited man,” “intersex,” “does not identify,” and “other” responses in Positive Plus One data

^e^ Sexual orientation data were not available from PHAC data. Exposure categories from PHAC data were used instead

^f^ This category includes men who identified as “gay,” “bisexual,” “two-spirited,” or “transsexual man who had sex with men,” “transsexual man,” “two-spirited man” in Positive Plus One data. This category also includes participants who identified as “gay,” “bisexual,” “queer,” or “two-spirited” and who were male in Positive Plus One data

^g^ This category includes “IDU” and “heterosexual contact” from PHAC data

^h^ This category includes “MSM” and “MSM/IDU” from PHAC data

^i^ This category includes “blood/blood products,” “other,” “no identified risk,” and “not reported” from PHAC data

^j^ For all provinces and territories for PHAC data, race/ethnicity information were not available before 1998 and race/ethnicity data were not available for Ontario before 2009

^k^ This category includes “Aboriginal” from PHAC data

^l^ For PHAC data, race/ethnicity information was not submitted by Quebec or British Columbia

### Relationship characteristics

Partners in current relationships were linked together to estimate dyad-level characteristics of each couple. If only one partner participated, we inferred relationship-level characteristics based on the index partner’s report of their primary partner’s characteristics. We compared the relationship-level characteristics of dyads and relationships where only one partner participated to examine differences between these groups. Complete dyads had longer relationship duration (mean:8.5, SD:8.3 v. mean:6.0, SD:7.7, *p* = 0.002), higher relationship satisfaction (mean:4.4, SD:0.5 v. mean:4.0, SD:0.9, *p* < 0.001), and were more likely to be virally suppressed (86% v. 77%, *p* = 0.043) compared to relationships where only one partner participated in the study (Table 4). A greater proportion of participants who were the sole representative of their relationships had missing or unknown viral suppression of the HIV-positive partner compared with complete dyads (13% v. 7%, *p* = 0.043).

**Table 4:** Relationship-level characteristics of dyads and individually represented HIV-serodiscordant relationships in PP1

Relationship characteristic	Total relationships (***N*** = 460) N (%)	Dyad (***n*** = 153) n (%)	Individual representative (***n*** = 307) n (%)	***p***-value
**Language (survey completion)**	Unavailable		Unavailable	–
Both English		138 (90.2)		
Both French		9 (5.9)		
English and French		6 (3.9)		
**Partner gender**				0.062
Male-male	221 (48.6)	85 (55.6)	136 (44.3)	
Male-female	213 (46.3)	62 (40.5)	151 (49.2)	
Other	26 (5.7)	6 (3.9)	20 (6.5)	
**Age difference between partners (HIV-positive – HIV-negative)**	**435**	**153**	**282**	0.268
Mean (SD)	−0.3 (9.8)	0.4 (9.5)	− 0.7 (9.9)	
Median (range)	0 (− 33, 34)	0 (− 29, 34)	0 (− 33, 34)	
**Race**	Unavailable		Unavailable	–
Both partners White		83 (54.2)		
Both partners not White		20 (13.1)		
Mixed		46 (30.1)		
One or both partners missing		4 (2.6)		
**Region**				0.339
British Columbia	49 (10.7)	20 (13.1)	29 (9.4)	
Prairies	65 (14.1)	20 (13.1)	45 (14.7)	
Ontario	265 (57.6)	84 (54.9)	181 (59.0)	
Quebec	51 (11.1)	15 (9.8)	36 (11.7)	
Atlantic	25 (5.4)	12 (7.8)	13 (4.2)	
Split across 2 regions	2 (0.4)	2 (1.3)	0 (0)	
Missing	3 (0.7)	0	3 (1)	
**HIV diagnosis or relationship first**	Unavailable		Unavailable	–
HIV diagnosis first		102 (66.7)		
Relationship first		38 (24.8)		
Same time		13 (8.5)		
**Relationship duration (years)**	**460**	**153**	**307**	**0.002**
Mean (SD)	6.8 (8.0)	8.5 (8.3)	6 (7.7)	
Median (range)	4 (0, 40)	5 (0, 40)	3 (0, 40)	
**HIV-positive partner virally suppressed**^**b**^				**0.043**
Yes	367 (79.8)	132 (86.3)	235 (76.5)	
No	43 (9.3)	11 (7.2)	32 (10.4)	
Don’t know	31 (6.7)	6 (3.9)	25 (8.1)	
Missing	19 (4.1)	4 (2.6)	15 (4.9)	
**Relationship Satisfaction – mean of partners**^**c**^	**458**	**153**	**305**	**< 0.001**
Mean (SD)	4.1 (0.8)	4.4 (0.5)	4 (0.9)	
Median (range)	4.3 (1, 5)	4.4 (2.4, 5)	4.1 (1, 5)	
**Sexual Satisfaction – mean of partners**^**c**^	**452**	**153**	**299**	0.799
Mean (SD)	3.7 (1.1)	3.8 (1)	3.7 (1.2)	
Median (Range)	4 (1, 5)	4 (1, 5)	4 (1, 5)	

Abbreviations: *PP1* Positive Plus One Study

^a^ Unavailable for couples with only one participating partner

^b^ For dyads, we used the response of the HIV positive partner where it was divergent from the negative partner’s response. HIV-positive participants in a past relationship who reported being suppressed at any time in the relationship were classified as suppressed while HIV-positive past-relationship participants who reported that they were virally suppressed “none of the time” during the relationship were counted as not suppressed

^c^ Possible range 1–5; (1 = low, 5 = high)

### Correlates of dyad participation

Among current index partners, 78% intended to invite their primary partner to take part in the study and 40% successfully recruited them. Index participants who were satisfied with their relationship were more likely to indicate that they intended to invite their HIV-serodiscordant partner to the study (81% v. 66%, *p* = 0.015) and were also more likely to have their partner enrol (42% v. 25%, *p* = 0.015) compared to those who were not satisfied with their relationship (Table 5).

**Table 5:** Recruitment and participation of primary HIV-serodiscordant partner by PP1 index partners in current HIV-serodiscordant relationships

Demographic characteristic	1st partner enrolled in study N (%)	Proportion intending to invite partner to the study^**a**^ % (95% CI)	***p***-value	Proportion of partners recruited to the study^**b**^ % (95% CI)	***p***-value
**Total (N)**	**387**	**303/387**	**–**	**153/387**	**–**
**HIV status**	**387**	**303**	0.335	**153**	0.088
Positive	250 (64.6)	76.8 (71.6–82.0)		36.4 (30.4–42.4)	
Negative	137 (35.4)	81.0 (74.4–87.6)		45.3 (36.9–53.6)	
**Language**	**387**	**303**	0.902	**153**	0.293
English	356 (92.0)	78.4 (74.1–82.6)		38.8 (33.7–43.8)	
French	31 (8.0)	77.4 (62.7–92.1)		48.4 (30.8–66.0)	
**Age**	**387**	**303**	0.125	**153**	0.855
18–29	50 (12.9)	68.0 (55.1–80.9)		38.0 (24.5–51.5)	
30–39	126 (32.6)	81.0 (74.1–87.8)		38.1 (29.6–46.6)	
40–49	97 (25.1)	83.5 (76.1–90.9)		43.3 (33.4–53.2)	
≥ 50	114 (29.5)	75.4 (67.5–83.3)		38.6 (29.7–47.5)	
**Gender**	**386**	**303**	0.172	**153**	0.875
Female	265 (68.7)	84.2 (77.5–90.9)		37.7 (28.8–46.6)	
Male	114 (29.5)	75.8 (79.7–81.0)		40.4 (34.5–46.3)	
Other^c^	7 (1.8)	85.7 (59.8–100.0)		42.9 (6.2–79.5)	
**Sexual orientation**	**384**	**302**	**0.068**	**153**	0.366
Heterosexual	150 (39.1)	82.7 (76.6–88.7)		37.3 (29.6–45.1)	
Lesbian/Gay	190 (49.5)	76.8 (70.8–82.8)		43.7 (36.6–50.7)	
Bisexual	25 (6.5)	84.0 (69.6–98.4)		36.0 (17.2–54.8)	
Other^d^	19 (5.0)	57.9 (35.7–80.1)		26.3 (6.5–46.1)	
**Race/ethnicity**	**380**	**300**	0.891	**151**	0.155
White	252 (66.3)	80.2 (75.2–85.1)		44.4 (38.3–50.6)	
Black	28 (7.4)	75.0 (59.0–91.0)		32.1 (14.8–49.4)	
Indigenous	36 (9.5)	77.8 (64.2–91.4)		25.0 (10.9–39.1)	
Hispanic	29 (7.6)	75.9 (60.3–91.4)		24.1 (8.6–39.7)	
East/SE Asian	11 (2.9)	72.7 (46.4–99.0)		36.4 (7.9–64.8)	
S Asian/W Asian/Arab	9 (2.4)	66.7 (35.9–97.5)		44.4 (12.0–76.9)	
Other^e^	15 (4.0)	87.7 (69.5–100.0)		40.0 (15.2–64.8)	
**Region**	**384**	**303**	0.647	**153**	0.673
British Columbia	45 (11.7)	86.7 (76.7–96.6)		46.7 (32.1–61.2)	
Prairies	51 (13.3)	78.4 (67.1–89.7)		39.2 (25.8–52.6)	
Ontario	224 (58.3)	77.7 (72.3–83.1)		37.9 (31.6–44.3)	
Quebec	40 (10.4)	75.0 (61.6–88.4)		37.5 (22.5–52.5)	
Atlantic	24 (6.3)	83.3 (68.4–98.2)		50.0 (30.0–70.0)	
**Relationship satisfaction**	**385**	**303**	**0.015**	**153**	**0.015**
Yes	332 (86.2)	80.7 (76.5–85.0)		42.2 (36.9–47.5)	
No	53 (13.8)	66.0 (53.3–78.8)		24.5 (12.9–36.1)	
**Sexual satisfaction**	**380**	**299**	0.711	**150**	0.298
Satisfied	248 (65.3)	79.0 (74.0–84.1)		41.5 (35.4–47.7)	
Neutral	67 (17.6)	80.6 (71.1–90.1)		31.3 (20.2–42.5)	
Dissatisfied	63 (16.6)	76.2 (65.7–86.7)		41.3 (29.1–53.4)	
Don’t know	2 (0.5)	50.0 (0 -100.0)		0	
**Diagnosis first or relationship first**	**387**	**303**	0.321	**153**	0.480
Diagnosis first	194 (50.1)	78.4 (72.6–84.1)		38.1 (31.3–45.0)	
Relationship first	109 (28.2)	74.3 (66.1–82.5)		37.6 (28.5–46.7)	
Same time	84 (21.7)	83.3 (75.4–91.3)		45.2 (34.6–55.9)	
**Viral suppression**	**373**	**294**	0.754	**149**	0.211
Undetectable	315 (84.5)	78.4 (73.9–83.0)		39.7 (34.3–45.1)	
Detectable	32 (8.6)	78.1 (63.8–92.4)		31.3 (15.2–47.3)	
Don’t know	26 (7.0)	84.6 (70.7–98.5)		53.8 (34.7–73.0)	

Abbreviations: *PP1* Positive Plus One Study

^a^ The proportion of current index partners enrolled in the Positive Plus One study who invited their partner to take part in the study

^b^ The proportion of current index partners enrolled in the Positive Plus One study who successfully recruited their partner to take part in the study

^c^ This category includes participants who identified as “trans man,” “trans woman,” “two-spirited man,” or “did not identify as any gender”

^d^ This category includes participants who identified as “queer,” “two-spirited,” “heteroflexible,” or “did not identify as any sexual orientation”

^e^ This category includes participants who identified as “other” (the most common other response was “mixed”)

### Missing responses

Self-completed surveys often include a large amount of missing/incomplete data, which may lead to potential biases if participants with missing data differ from participants with complete data [45]. The proportion of missing and “don’t know” survey responses were low (mean: 3%, median: 2% per survey) and a comparison of missing data patterns throughout the survey found that respondent fatigue was not an issue [34]. The proportion of missing and/or “don’t know” survey responses to sexual behaviour questions such as frequency of sex with partner, sex with other partners during relationship, condom use during intercourse, and sexual satisfaction ranged from 0.3–2%. There was also a low proportion of missing and/or “don’t know” survey responses to potentially sensitive survey questions such as income (2%), disclosure of HIV-serodiscordant relationship status to physician (2%), disclosure of HIV-serodiscordant relationship status to anyone outside of relationship (1%), and injection drug use (1%). Questions with the highest non-response included the sensitive question about abuse in the relationship (4%) and HIV-positive partner’s last viral load measure (3%), according to the positive partner.

## Discussion

Led by a large diverse group of investigators, PP1 provided key insight on the use of a mix of in-person and online strategies to recruit a diverse sample of individual HIV-positive and HIV-negative partners and dyads involved in a current or past HIV-serodiscordant relationship in Canada from 2016 to 2018. We found that main sources of recruitment varied between HIV-positive and HIV-negative partners. Additionally, the majority of index partners were willing to recruit their primary partner to the study and 40% were successful. Given differences in characteristics between recruited dyads and relationships where only one partner was enrolled in the study and between individuals involved in a current or past HIV-serodiscordant relationship, our findings support the need for the inclusion of both dyads and individual partners involved in a HIV-serodiscordant relationship and those involved in both current and past HIV-serodiscordant relationships to gain a full understanding of the experiences of living in a HIV-serodiscordant relationship.

Due to the hidden nature of HIV-serodiscordant relationships, our study used active recruitment by ASO staff and clinicians when possible and passive recruitment when staffing resources were not available. While we recruited the majority of our participants through staff or physicians at HIV clinics and/or ASOs, a large portion of our participants learned of the study through poster, pamphlet, or cards displayed at these and other recruitment sites and through their partner and/or friends. Among those involved in a current HIV-serodiscordant relationship, HIV-positive participants were more likely to learn of our study through HIV clinics and/or ASOs via physicians, staff, and/or newsletters whereas HIV-negative participants were more likely to learn of our study through their partner. These findings suggest that our multi-pronged approach to participant recruitment was needed to recruit HIV-positive and HIV-negative individuals involved in different types of HIV-serodiscordant relationships. Since the majority of HIV-negative individuals heard about our study through their partner, the PP1 recruitment strategy expanded as the study progressed to include the placement of posters and pamphlets at pharmacies dispensing PrEP, anonymous HIV testing sites, and methadone clinics. Ultimately these were effective at reaching HIV-negative individuals.

Similar to a study conducted by Starks and colleagues, we found that a high proportion of index participants (78%) were willing to invite their primary HIV-serodiscordant partner to participate, and 40% of these index participants successfully recruited their primary partner to enrol [30]. Following the lessons learned from previous studies, each partner was asked to complete the survey independently to limit coercion and partner bias and increase the participation of both partners in the dyad. We found that dyads provide more complete information compared individual partners on some variables of interest. For example, 13% of individuals who were the sole representative of their relationship reported the viral suppression status of the HIV-positive partner in the relationship as “unknown” or “missing” compared to 7% of dyads. However, viral suppression reported by dyads may result from healthier relationships and therefore may also be biased towards healthier outcomes. Additionally, studies examining HIV-serodiscordant relationships that only examine dyads may be biased towards those with higher relationship and/or sexual satisfaction (i.e., “happy couple” bias). Participants with higher relationship satisfaction and longer relationship duration were more likely to successfully engage their partner to take part in the study. Previous dyadic studies conducted among gay and heterosexual couples have noted that their samples included disproportionately more satisfied couples and couples with more confidence in their relationship [29–31]. More creative strategies are needed to help index partners recruit their partner in relationships with lower satisfaction and shorter duration as these populations may be under-represented in the current study and may be in need of different types of support.

Our study was not designed to capture detailed information on polyamorous HIV-serodiscordant relationships. A previous study among individuals involved in a hierarchical polyamorous relationship reported lower relationship satisfaction with their secondary and tertiary partners compared to non-hierarchical and primary partners [46]. While polyamorous partners were eligible to participate in the study, we did not ask whether their relationship was polyamorous, and could only link one serodiscordant partner to an index partner. Additional serodiscordant partners completed the survey as unlinked individuals. Future studies may wish to expand their inclusion criteria to individuals involved in casual relationships and expand analytic approaches to incorporate polyamorous relationships. Longitudinal studies are needed to observe the impact of changes in relationship satisfaction and sexual behaviour over time and correlates of relationship termination, an outcome of interest for HIV-serodiscordant couples. While PP1 tried to capture these outcomes retrospectively, the cross-sectional design limited our ability to draw firm conclusions.

PP1 has provided insights into several challenges of a multi-pronged approach for recruiting individuals involved in current or past HIV-serodiscordant relationships. Although we used a multi-pronged recruitment approach, the majority of our sample was recruited from ASOs and/or clinics, which may have led to potential biases in our sample. A previous study conducted in Ontario, Canada revealed that individuals living with HIV who used ASOs were less healthy, had lower quality of life, and lower income compared to those who did not receive services from ASOs [47]. Hence, our study may have over-represented individuals involved in HIV-serodiscordant relationships with poorer health and lower quality of life and income. Additionally, recruitment via physicians and clinics may have introduced bias towards those who were more connected to HIV care, and therefore, ART initiation and viral suppression [48]. Despite our best efforts to reach as many HIV-serodiscordant couples as possible, a comparison with the PHAC national HIV surveillance database indicated that our sample under-represented HIV-positive individuals who were ≥ 30 years of age at the time of diagnosis, men, those self-identifying as Black or Indigenous, and those who resided in British Columbia, Alberta, Quebec, and the three territories. The under-representation of Black or Indigenous HIV-positive individuals in our study may indicate that we were unable to adequately reach these populations despite our efforts to engage recruitment sites that work with them. These populations are generally known to be underrepresented in research studies [32]. Previous studies have shown that internet-based recruitment strategies including Facebook advertising can be a cost-effective method of recruiting a diverse sample of participants who are at risk of acquiring HIV infection, particularly those identifying as racial minorities [36, 37]. PP1 only recruited a small proportion of participants through online ads (e.g., Facebook), which could explain under-recruitment. Additional discussions with key stakeholders may be necessary to improve participation among these populations [32]. It may also be possible that HIV-positive individuals who were underrepresented in PP1 relative to national surveillance data are less likely to be involved in HIV-serodiscordant relationships compared to other HIV-positive individuals. However, since HIV-serodiscordant relationship status is frequently unknown to clinicians and ASO staff and there is a lack of a routine database of individuals involved in HIV-serodiscordant relationships in Canada, these findings are difficult to confirm. It should also be noted that PHAC data may not be representative of the current population living with HIV as it includes individuals who were diagnosed since the beginning of the HIV epidemic, a large proportion of whom identified as White and MSM and may no longer be alive [42]. As such, PHAC data may have over-represented the proportion of men living with HIV, partially explaining the lower proportion of men living with HIV enrolled in PP1 compared to PHAC surveillance data.

Our study did not have a budget to reimburse ASOs, NGOs, and clinic staff for their time spent actively recruiting participants. When organizations were initially approached about involvement in recruitment, many asked about cost recovery for recruitment activities. As a result of governmental ASO funding cutbacks during the course of study recruitment, active recruitment likely decreased as the study progressed [49]. Without a dedicated budget for recruitment efforts, accessing hard-to-reach populations likely worsened over time and overall recruitment took longer than expected.

Finally, PP1 took place in a Canadian setting, and sought to recruit individuals involved in a primary HIV-serodiscordant relationship. As such, findings may be generalizable to resource-rich settings.

## Conclusions

Our findings provide important insights that support the use of a multi-pronged approach to recruit a diverse sample of individuals involved in current or past HIV-serodiscordant relationships in Canada that include a mix of complete dyads and lone participants in current HIV-serodiscordant relationships. Our findings suggest several considerations to facilitate the recruitment, enrollment, and engagement of a diverse sample of individuals involved in HIV-serodiscordant relationships for future studies. In particular, the recruitment of both dyads, lone participants, and individuals involved in past HIV-serodiscordant relationships can help researchers obtain a more diverse sample in terms of relationship duration and satisfaction. Additionally, future studies should employ creative strategies to recruit individuals involved in past, casual, and/or polyamorous HIV-serodiscordant relationships and relationships with lower satisfaction in order to further minimize the risk of “happy couple” bias. Future studies that focus on hard-to-reach areas and populations should involve more discussion with stakeholders, expand the use of social media platforms, and approach more sites utilized by HIV-negative individuals (e.g., pharmacies prescribing PrEP, anonymous HIV-test sites, methadone clinics) to increase enrollment from underrepresented populations to ensure the recruitment of a more representative sample. Budgeting for active recruitment in ASOs and clinics where staffing may be limited is essential to increase enrollment.

## Data Availability

The datasets generated and/or analysed during the current study are not publicly available due to the inclusion of potentially identifying and sensitive information but are available from the corresponding author on reasonable request.

## Abbreviations

ART: Antiretroviral therapy
ASO: AIDS services organization
CPHSP: Canadian Perinatal HIV Surveillance Program
HASS: HIV/AIDS Surveillance System
HIV: Human immunodeficiency virus
IP: Internet protocol
IRCC: Immigration, Refugees and Citizenship Canada
MSM: Men who have sex with men
NGO: Non-governmental organization
PEP: Post-exposure prophylaxis
PHAC: Public Health Agency of Canada
PP1: Positive Plus One
PrEP: Pre-exposure prophylaxis
SD: Standard deviation
U=U: Undetectable = Untransmittable

## Footnotes

1 Survey software was developed and hosted by Demographix Ltd.
